# Phytochemical Profile, Antioxidant Activity, Anti-Hyperglycemic Effect and Toxicity Assessment of *Ridolfia segetum* (L.) Moris Extract

**DOI:** 10.3390/life13010044

**Published:** 2022-12-23

**Authors:** Jamila El Karkouri, Amale Kchibale, Mounia Chroho, Brahim Eddamsyry, Hanane Touijer, Fadoua El Makhoukhi, Nadia Handaq, Bruno Eto, Ahmad Mohammad Salamatullah, Mohammed Bourhia, Touriya Zair

**Affiliations:** 1 Research Team of Chemistry of Bioactive Molecules and the Environment, Laboratory of Innovative Materials and Biotechnology of Natural Resources, Faculty of Sciences, Moulay Ismaïl University, B.P. 11201 Zitoune, Meknes 50070, Morocco; 2 Laboratoires TBC, Laboratory of Pharmacology, Pharmacokinetics and Clinical Pharmacy, Faculty of Pharmacy, University of Lille, 3, Rue du Professeur Laguesse, B.P. 83, F-59000 Lille, France; 3 Department of Food Science & Nutrition, College of Food and Agricultural Sciences, King Saud University, 11 P.O. Box 2460, Riyadh 11451, Saudi Arabia; 4 Laboratory of Biochemistry, Faculty of Medicine and Pharmacy, Laayoune 70000, Morocco

**Keywords:** *Ridolfia segetum* (L.) Moris, polyphenols, DPPH, FRAP, toxicity, antihyperglycemic

## Abstract

The present work was designed to study the chemical composition, antioxidant, antihyperglycemic effect, and toxicity assessment of *Ridolfia segetum* (L.) Moris extract. The chemical composition was studied by use of high-performance liquid chromatography (HPLC). Antioxidant power was tested by use of DPPH and FRAP assays. The antihyperglycemic effect was tested by use of a glucose tolerance test, while toxicity assessment was done in vivo by use of Wistar rats for 14 days. Analysis of the extract by HPLC-UV revealed the presence of gallic acid, catechol, vanillic acid, catechin, tannic acid, rosmarinic acid, naringenin, and coumarin acid. The crude hydroethanolic extract possessed high levels of total phenols (15.6 ± 1.76 mg EAG/g), condensed tannins (383.49 mg ECat/g DM), and flavonoid (11.63 mg EQ/g). The findings showed that the studied extract possessed good antioxidant power with IC_50_ values equal to 550, 650, 700 µg/mL respectively for the decoction, the ethyl acetate fraction (F_2M_), and the ethyl acetate fraction (F_2E_). For the antioxidant activity by FRAP, the aqueous fraction (F_3E_) and the aqueous extract (F_4_) showed CE_50_ values of 0.33 mg/mL and 0.4 mg/mL, respectively. Glucose tolerance test analysis showed that *R. segetum* (L.) Moris decoction had a significant postprandial antihyperglycemic effect in normal Wistar rats. The results of the acute toxicity test showed that the decoction was not toxic even at 2 g/Kg. Pancreatic α-amylase activity was significantly inhibited in the presence of *R. segetum* (L.) Moris extract (IC_50_ = 0.133 ± 0.09 mg/mL). The outcome of the present work showed that *R. segetum* (L.) Moris is very rich in phenolic compounds with potent antioxidant and antihyperglycemic effects.

## 1. Introduction

For a long time, thorough scientific research has focused on the search for new therapeutic molecules of natural origin. This trend can be largely explained by the pressing need for new disease treatments. Aromatic and medicinal plants (MAP) are a very important source to get new, valuable, and amazing chemical molecules. These compounds are often metabolized into active substances with distinct identities according to the qualities they possess, for example, the treatment or cure of many chronic diseases such as diabetes. *Ridolfia segetum* (L.) Moris is a plant distributed in the Mediterranean, extending as far as Anatolia and Macaronesia, and weed in Western and Central Europe [1] where it was introduced in particular with cereal grains [2]. This plant grows throughout Morocco, especially on clay soils [3]. It is a glabrous, annual plant of 40–80 cm. It belongs to the Apiaceae family; it is easily recognizable by its tripinnate leaves with filiform division, elongated, and divaricate. The upper leaves are reduced to the expanded sheath. The flowers are yellow, in umbels of 10 to 40 slender rays, almost equal *R. segetum* (L.) Notably. Traditional medicine makes extensive use of Moris for treating a broad variety of illnesses, including: cough, constipation, respiratory tract infections, and stomach acidity. [4,5,6]. It regulates menstruation in women and increases milk production in nursing mothers, the infusion of grains helps to fight against constipation and intestinal gas [7]. It is a plant eaten by livestock, especially before flowering [8]. The ethnobotanical studies in Morocco have indicated the traditional use of *R. segetum* (L.) Moris as an antidiabetic plant [9,10,11]. The frequent use of this species in traditional medicine and the confirmation of its effectiveness by scientific laboratory studies have led to the identification of its various biological properties such as antioxidant activity [5,6], antibacterial [6,12,13,14], anti-inflammatory [5], anticancer [15,16], HIV-1 inhibition [4] and insecticidal activity [14,17]. Diabetes mellitus is defined by persistent hyperglycemia, which affects carbohydrate, lipid, and protein metabolism and comes from a deficiency in the production of insulin, the action of insulin, or both conjugates. [18]. The symptoms of diabetes mellitus are not visible; however, the presence of hyperglycemia permanently causes progressive disturbances in the capillaries and the appearance of long-term complications affecting in particular the eye, the kidneys, nervous and cardiovascular systems [18]. This is why a diagnosis of the disease is necessary for early detection and establishment of adequate treatment. An estimated 80% of people worldwide utilize medicinal plants as a primary or secondary method of illness treatment, according a recent World Health Organization research. When it comes to diabetes treatment, over 1200 different medicinal plants are employed, coming from over 725 different genera and 183 different families. [19]. In addition, 81% of antidiabetic plants used in traditional medicine gave positive results and showed a reduction in hyperglycemia [20]. The present work is interested in the phytochemical study, the evaluation of the antioxidant power, the toxicity, the anti-hyperglycemic effect, and the inhibitory activities of the pancreatic α-amylase of the extracts of *R. segetum* (L.) Moris in order to confirm the medicinal uses of this species.

## 2. Material and Methods

### 2.1. Plant Material

#### 2.1.1. Harvest and Botanical Identification

**Figure 1:**
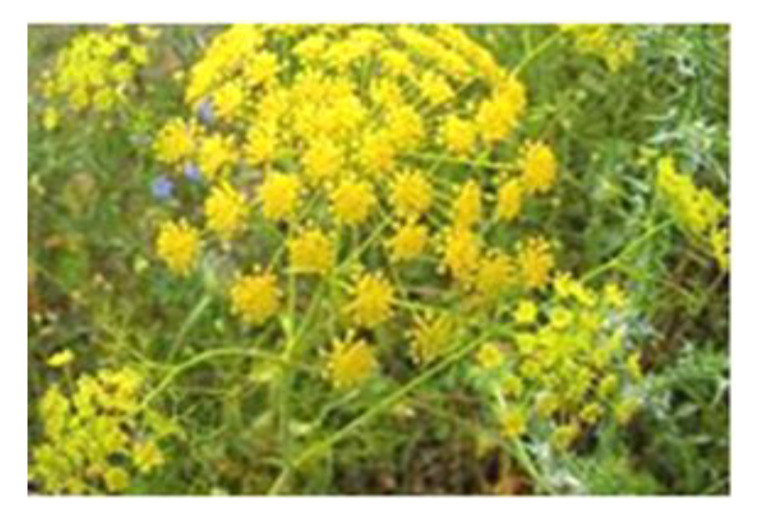
*Ridolfia segetum* (L.) Moris.

*R. segetum* (L.) Moris RAB114053 was harvested in June 2019 at the time of its flowering in the rural commune Dar Algadari, Kenitra—Morocco. The botanical identification was conducted at the the Scientific Institute of Rabat. Importantly, *R. segetum* (L.) Moris belongs to the family Apiaceae according to Coste, H et al. [21] (Table 1, Figure 1).

#### 2.1.2. Quality Control of Plant Material

##### pH Determination

For pH measurement, 10 mL of distilled water was added to 2 g of the material being tested. The mixture was agitated before being filtered and chilled. The pH value was then determined by immersing the electrode in a significant amount of the filtrate.

##### Content of Mineral Matter (Ash) and Organic Matter

The method used is that described by the AFNOR 1977 [22]. After the annihilation of organic matter by incineration, the raw ashes was recovered. After drying in an oven for an hour, nickel crucibles are placed in a desiccator before being weighed. Each crucible was then tared to within 0.1 mg when it has cooled. Three grams of material are reduced to a powder and then weighed in crucibles to the closest 0.1 milligram. After filling the crucibles, they were placed in the muffle furnace with the extractor hood above them, where they will be cal-cined for four hours at 550 °C. In the end, we waited for the temperature to drop to 100 degrees Celsius, removed the crucibles, let them cool in a desiccator, and then weighed them to the closest 0.1 g. To determine the overall amount of ashes, one uses the formula:

$$\mathrm{MM}\%=\left(\frac{M_{f}-M_{0}}{\mathrm{TS}}\right)*100$$

MM: Mineral Matter (Ash Content) expressed as a percentage of gross product.

M_0_: Mass of the empty crucible, in grams.

M_f_: Mass of the crucible containing the dry residue, in grams

TS: Mass of the test sample, in grams

The organic matter content MO of the sample is the difference between the mass of matter solids MS and the mass of mineral matter expressed as a percentage according to the following relationship:

MO=MS−MM 

##### Dry Matter Moisture Content

Drying a sample at 103 °C for 24 h results in a loss of mass proportional to the amount of moisture present in the sample. To do so, 5 g of sample (for the six plants) was weighed, then put in crucibles that have been dried, tared, and oven-ready at 103 °C for 24 h. They are then placed in a desiccator to cool down and then weighed once this time period has passed. When the AFNOR standard NF V 18-1095 was followed, the result is given as a percentage of dry matter..

$$\mathrm{TH}\%=\frac{m_{1}-m_{2}}{m_{1}}*100$$

m_1_ = Number of grams of vegetables at the outset placed in the oven (t0).

m_2_ = Plants’ final mass in grams after being taken out of the oven (tx).

The rate of dry matter is expressed:

$$\mathrm{MS}\%=\frac{M_{f}-M_{0}}{\mathrm{TS}}*100$$

M_0_: Mass of the empty crucible, in grams.

M_f_: Mass of the crucible containing the dry residue, in grams.

TS: Mass of the test sample, in grams.

##### Determination of Mineral Composition by ICP-AES

The induc-tively coupled plasma (ICP) spectrometry method was used to identify the presence of trace elements, heavy metals, or metallic trace elements. This technique makes an effort to get as near as feasible to the quantity of usable mineral elements present in the plant. Its minor and major mineral compositions were quickly and precisely determined using inductively coupled plasma spectrometry. At 110 degrees Celsius, 1 g of the plant powder was heated in a solution of 5 milliliters of strong nitric acid HNO_3_ and 15 milliliters of hydrochloric acid. Upon completion of the solubilization process, the sample was brought down to room temperature and diluted with ultrapure water to a final volume of 100 mL. We next used ICP-AES to directly assess the amounts of trace metals in the solution, doing this analysis in triplicate (Agilent 5110 ICP-OES Spectrometer). The stable plasma gas flow is kept at 15 L/min, while the auxiliary gas flow is kept at 0.2 L/min and the nebulizer gas flow is kept at 0.8 L/min. 1.5 mL/min of sample flow was achieved at 1500 W of power.

### 2.2. Phytochemical Study

#### 2.2.1. Phytochemical Screening

Various classes of chemicals present in the plant was screened by conducting qualitative characterisation processes. These are precipitation or staining processes that need reagents unique to each class of chemicals [23,24,25].

#### 2.2.2. Extraction of Polyphenols

##### Decoction Extraction

The extraction method was done by placing 30 g of the powder and 600 mL of distilled water in the Erlenmeyer flask, the mixture was heated (reflux system) for one hour then filtered using filter paper and concentrated under reduced pressure, then dried in an oven at 40 °C. The decoction is kept until it is used.

##### Soxhlet Extraction

A sample of 30 g of powdered R was subjected to extraction by soxhlet, a method that employs the use of solvents in the liquid phase for the purpose of treating plant material. A cartridge containing segetum (L.) Moris was inserted into the soxhlet’s extraction chamber. Extracted plant material was then subjected to a second extraction procedure, this time employing water as the solvent for the soxhlet extraction. Depletion of plant stuff requires many cycles. After filtering, a rotary evaporator is used to remove the remaining solvent at temperatures as low as 50 °C and decreased pressure. Hydroalcoholic and aqueous extracts of crude polyphenol residues are thus produced.

##### Splitting

The plant powder was extracted using methanol, ethanol, ethyl acetate and n-butanol, which have a different polarity. Next, Moris extracts were kept at 4 °C until they were used.

#### 2.2.3. Amount of Total Phenols

Singleton [26] reported the use of the Folin-Ciocalteu reagent in the measurement of total polyphenols in 1965 [27]. Briefly, two microliters of each extract were put to a 100 mL volumetric flask, followed by tenfold dilutions of Folin-reagent Ciocalteu’s (1.5 mL) and 7.5% sodium carbonate (1.5 mL). After filling the vials with distilled water, they wereshaken and allowed to sit at room temperature for 30 min. Use a spectro-photometer to compare the sample to a blank absorbance reading taken at 760 nm. The production of a calibration curve using gallic acid as a positive control was carried out in parallel under identical working conditions. The findings were reported in milligrams (mg) of gallic acid equivalent per gram of dry plant material (mg GAE/g). The total phenol content is computed using the formula:

$$T=\frac{C*V_{0}}{\;m_{extrait}\;}*D$$

With C: Concentration evaluated according to the calibration curve. 

V_0_: Volume of the overall extract.

$$D=V_{f}/V_{i}$$

With D: Dilution factor.

V_f_: Final volume to be measured by spectrophotometer.

Vi: Sample volume of the extract to be tested.

#### 2.2.4. Amount of Flavonoids

The flavonoid contents are measured using aluminum trichloride [28], the latter forms with the flavonoids a yellow complex which absorbs in the visible at 433 nm. The blank is produced by replacing the extract with methanol and the absorbance is measured at 433 nm. 2 µL of each extract is mixed with 10 µL of 10% (*m*/*v*) aluminum trichloride (AlCl_3_), followed by 2 mL of distilled water and 3 mL of pure methanol. After 30 min incubation at room temperature, absorbance is determined at 433 nm using a UV spectrophotometer against a blank. A calibration curve is produced in parallel under the same operating conditions using quercetin as a positive control. The flavonoid content of the different fractions are expressed in milligrams (mg) the equivalent of quercetin per gram of dry plant matter (mg EQ/g). The flavonoids content is calculated using the formula:

$$T=\frac{C*V_{0}}{\;m_{extrait}\;}*D$$

#### 2.2.5. Amount of Condensed Tannins

The concentration of condensed tannins was measured in an acid medium using the vanillin technique [29]. Different quantities of (+)-catechin solution (2 mg/mL) are mixed with 3 mL of vanillin/methanol solution (4% *m*/*v*). Each and every one of these mixtures was prepared by hand by a human. Each concentration was then given an additional 1.5 cc of strong hydrochloric acid. The resulting mixes need 20 min to react at room temperature. A spectrophotometer absorbance measurement was made at 499 nm and compared to a blank. Condensed tannins in our samples were counted by using the same procedure as when drawing the calibration curve, except we substituted catechin for our samples. The calibration curve is used to determine the tannic acid content in milligrams of catechin equivalents per gram of dry matter.

#### 2.2.6. High-Performance Liquid Chromatography Coupled with Diode Array Detector Analysis

In order to conduct a chromatographic analysis of the polyphenolic extracts of the plant under study, we used a Waters HPLC e2695 (Milford, MA, USA) with a UV/Vis detector. The dimensions of the column utilized are 5 m × 250 mm × 4.6 mm; it is a C18 reverse phase column. When eluting molecules, a combination of water/acetic acid (2% *v*/*v*) (A) and acetonitrile, pH = 2.6, is used as the mobile phase (B). This experiment’s solvent gradient goes as follows: 2% acetonitrile (isocratic) 0–3 min, 2% acetonitrile in ace-tic acid (linear gradient) 3–19 min, 30% acetonitrile in acetic acid (linear gradient) 19–23 min, 80% acetonitrile in acetic phosphoric acid (isocratic) 23–28 min, 80% acetonitrile in acetic One milliliter per minute is the current rate of flow. Ten microliters (L) of injection volume. A diode array detector was used for the detection, and its spectral response was optimized for the region of 280–360 nm. Gallic acid, lecatechol, vanillic acid, catechin, tannic acid, rosmarinic acid, naringenin, coumarinic acid, and naringenin are used as reference points. The retention periods of the various peaks of the extracts are compared to the retention times of the peaks corresponding to the standards, allowing for an analysis of the eluted chemicals.

#### 2.2.7. Antioxidant Activity

##### DPPH* Free Radical Scavenging

The capacity of antioxidants to scavenge the DPPH* radical is the basis of this approach [30]. Extraction of each sample fraction or standard antioxidant (Ascorbic acid) at varying concentrations (from 0 to 200 g/mL) was mixed with 2.8 mL of the DPPH solution from before. At the same time, 200 Ul of ethanol is combined with 2.8 mL of DPPH* solution in ethanol to make a negative control. After 30 min in the dark at room temperature, the absorbance is measured against a blank at 515 nm [31,32]. Under the same circumstances and at the same concentrations as the samples, the absorbance of a solution of a standard antioxidant, ascorbic acid, served as the positive control [33]. There will be three separate tests. Results were reported as a percentage of inhibition (PI%):

$$PI\%=\left[\frac{Abs_{control}-Abs_{test}}{Abs_{control}}\right]*100$$

With:

Abs _Control_: Absorbance Control

Abs _test_: absorbance of the test performed

IC_50_ or 50% Inhibitory Concentration is the concentration of the test sample needed to reduce 50% of DPPH radical*. CI_50_s are calculated graphically by linear regressions.

##### FRAP Iron Reducing Power Test

Each extract was combined with a 0.2 M phosphate buffer solution (pH = 6.6) and a 1% solution of potassium ferricyanide K3Fe (CN) 6 in a total volume of 5.0 mL. The whole solution was put into a water bath at 50 degrees Celsius for 20 min. After adding 2.5 mL of 10% trichloroacetic acid to halt the reaction and centrifuging the tubes at 3000 rpm for 10 min, the resulting supernatant is mixed with 2.5 mL of distilled water and 0.5 mL of an aqueous 0.1% FeCl3 solution. At 700 nm, the reaction medium absorbance is measured against a blank in which the extract has been replaced by distilled water. A standard antioxidant solution, ascorbic acid, was used as the positive control, with its absorbance measured in the same way as the samples. If the extracts’ absorbance increases, it means they have more reducing power [34,35].

### 2.3. Pharmacological Study

The evaluation of the pharmacological activity consists of highlighting an effect, quantifying this effect by studying the dose/response and response/time relationships, looking for side effects and finally studying the mechanism of action. The prepared extracts are subjected to preliminary pharmacological tests in vitro or in vivo in order to verify their use in traditional medicine.

#### 2.3.1. Animals

The mice were reared under controlled conditions (a photoperiod of 12 h of light/12 h of darkness, and a temperature of 22 2 °C) at the animal facility of the Biology Department at the Fac-ulty of Sciences of Oujda. The animals were housed in a clean, dry, and well-fed environment where breeding was encouraged. To determine toxicity, albino mice were used in the experiment (male and female)

#### 2.3.2. Acute oral Extract of *R. segetum* (L.) Moris

The objective of this test is to show that the therapeutic dose used is not toxic in the short term in normal mice. Two batches of albino mice (20–35 g) in the fasting state (14 h) were randomly divided into 4 groups (n = 6; ♂/♀ = 1).

Control: DE (10 mL/Kg).

Group 1: the decoction (0.5 g/Kg).

Group 2: the decoction (1 g/Kg).

Group 3: the decoction (2 g/Kg).

At the start of the test, the mice were weighed. Afterward, they were force-fed with a single dose of the decoction. Thereafter, we monitored them continuously for 10 h in order to note the signs of apparent toxicity. For the remaining time which is 14 days, the mice were monitored daily to notice if there were any additional clinical or behavioral signs of toxicity.

#### 2.3.3. Study of the Antihyperglycemic Effect of *R. segetum* (L.) Moris Extract in Normal Rats

**Figure 2:**
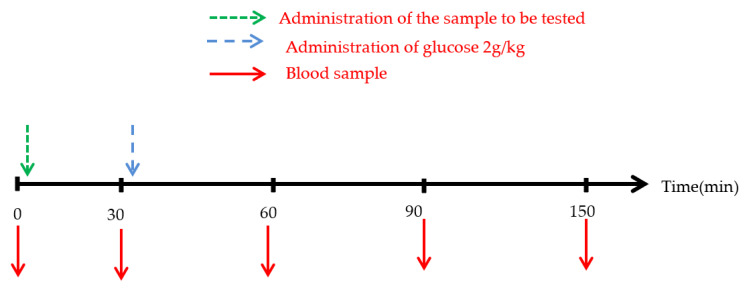
Timeline of oral glucose tolerance test in normal rats. Glib: Normal rats force-fed with glibenclamide (2 mg/mL).

This study aims to investigate whether these extracts exhibit a postprandial antihyperglycaemic effect in normal rats overloaded with D-glucose (Figure 2).

##### Oral Glucose Tolerance Test

To test the antihyperglycemic effect (postprandial blood glucose) of the plant extract in vivo, the oral glucose tolerance test was used. Normal rats were divided into three groups (n = 6; ♂/♀ = 1).

Control: Normal rats force-fed with ED (10 mL/Kg);

Decocted: Normal rats force-fed with the extract (400 mg/Kg);

##### Evaluation of the Inhibitory Effect of Aqueous Extracts on the Activity of Pancreatic α-Amylase, In Vitro

α-amylase, is an enzyme involved in the catabolism of long-chain carbohydrates into smaller units. These cause postprandial hyperglycemia. One reason why this study aimed to evaluate the inhibitory effect of *R. segetum* (L.) Moris extract on the activity of the α-amylase enzyme.

The inhibitory effect of the aqueous extract on the enzymatic activity of α-amylase is carried out according to the method described by Daoudi et al. A volume of 200 μL of the aqueous extract solution or the acarbose solution (positive control) is added to 200 μL of the phosphate buffer solution (0.02 M, pH = 6.9. A volume of 200 μL of the enzyme solution (α-amylase from porcine pancreas (APP) (E.C.3.2.1.1). α-amylase is in powder form of 10 IU/mg) was added to all tubes except the blank where the enzyme solution is replaced by phosphate buffer. The aqueous extract was tested using the following concentrations: 0.89, 0.45, 0.22, 0.11, and 0.06 mg/mL. Ascorbic acid was tested using the following concentrations: 1, 0.8, 0.6, 0.4, and 0.2 mg/mL.

Tubes were preheated to 37 degrees Celsius for 10 min. After that, 200 L of starch solution were added to each tube. After everything has been combined, it is incubated at 37 degrees Celsius for 15 min. The enzymatic process was terminated by adding 600 L of DNSA. The test tubes are then heated in a water bath at a rolling boil for 8 min. A thermal shock is then used to halt the reaction by immersing the tubes in an ice water bath before adding 1 mL of distilled water to each tube. The absorbance is measured at 540 nm using a spectrophotometer with a buffer solution serving as a blank. Inhibition percentages for each extract and acarbose may be determined using the following formula:

$$\%\;\mathrm{Inhibition}=\frac{\left[Ab_{\mathrm{control}\;}-Ab_{\mathrm{sample}}\right]}{Ab_{\mathrm{control}}}*100$$

Ab_control_: Absorbance of enzyme activity without inhibitor. Ab_sample_: Absorbance of enzymatic activity in the presence of extract.

## 3. Results and Discussion

### 3.1. Quality Control

The results relating to the quality control (pH, TH, ash, and ICP) are grouped in Table 1.

#### 3.1.1. pH Determination

From Table 2, we found that *R. segetum* (L.) Moris has a very weakly acidic pH of around 5.21.

#### 3.1.2. ASH Content

The results of the ash percentage are deduced according to the following formula:

% Ash = 100 − MO%. (OM: organic matter)

Leaves of green plants are where the organic matter is made, from a combination of water, carbon dioxide, and minerals. The percentage of organic matter was found to be equal to (2. 180 ±15)% after calcination at 550 °C in a muffle furnace till generating whit-ish ashes, whereas the percentage of ash was found to be equal to (97.820 ±11%). Organs like leaves and flowers, which contain significant percentages of mineral materials, demonstrate the significance of both organic and mineral constituent compositions. Soil mineral content, root absorption efficiency, and mineral transport to the plant’s leaves and stems all play a role in this variance.

#### 3.1.3. Dry Matter Rate and Humidity Rate TH (%)

Three samples of five (5) grams of vegetable matter are dried at 100 °C in an oven until a consistent weight is reached, and the resulting moisture content is determined to be in the range of (12.49 0.01)% (Table 2).

#### 3.1.4. Analysis of the Mineral Composition of *R. segetum* (L.) Moris by ICP/AES

R uses ICP/AES analysis to determine elemental mineral concentration. You can see the results for segetum in Table 3. Based on our findings, calcium is the most abundant element in our species at a concentration of around 47.685 mg/L, followed by iron at a value of approximately 0.672 mg/L. No traces of the Cd component exist.

Due to its high calcium, iron, aluminum, and manganese content and low levels of cadmium and copper, zinc, and arsenic, the species under study may be a good source of healthful, non-toxic food.

### 3.2. Phytochemical Screening

Screening for phytochemicals is a qualitative study that attempts to classify plant chemical families. In order to determine the phytochemical properties of the plant extracts, several revealing reagents are applied to the samples. Table 4 displays the outcomes of the experiments.

From the table of results, we find that *R. segetum* (L.) Moris is rich in sterols and triterpenes, in combined anthraquinones of the genin c-heteroside type, flavonoid glycosides, and catechol flavonoids, in gallic tannins, as it contains an average amount of mucilage. But, it is poor in alkaloids and devoid of leucoanthocyanins, saponosides, catechin tannins, oses and holosides and free antraquinones. These results are in agreement with those of El karkouri et al., Ben sahaKhira et Hasini Ahlam, Marongiu, et al. [35,36,37].

### 3.3. Extraction Yields of Polyphenols from R. segetum *(L.)* Moris

**Figure 3:**
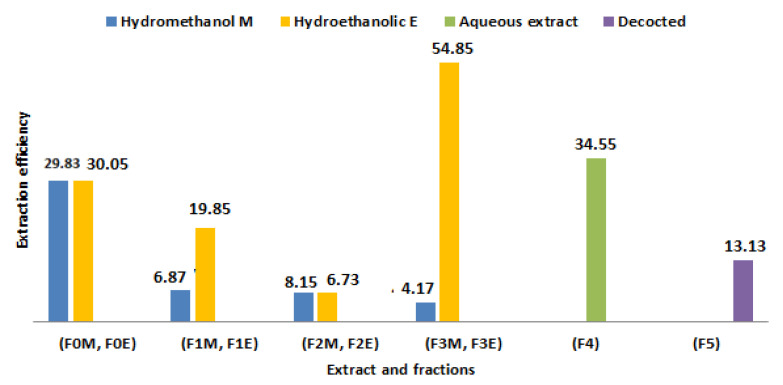
Yields (%) of *R. segetum* (L.) Moris extracts.

We carried out the solid-liquid extraction of *R. segetum* (L.) Moris by two extraction methods (Soxhlet and decoction) using several solvents of different polarities in order to compare the yields and the contents of the polyphenols. We obtained a total of ten extracts, the results of which are given in Figure 3; these are the crude hydrometanol (F_0M_) and hydroethanolic (F_0E_) extracts, the ethyl acetate fractions (F_1M_, F_1E_), the butanol fractions (F_2M_, F_2E_) and the aqueous fractions (F_3M_, F_3E_), l aqueous extract (F_4_) and the decoction (F_5_). We find that the raw, aqueous extracts and the residual fraction gave quite high yields, compared to the others, whose yields are respectively of the order of 30.05%; 29.83; 34.55 and 54.85%.

From Figure 3 it appears that the yields obtained by soxhlet for all the extracts studied are higher than those obtained by decoction, and the best extraction solvent is the hydroethanolic mixture.

These results are in agreement with numerous studies carried out on the extraction of phenolic compounds, which have shown that the release of bioactive compounds is favored by the use of mixed solvents such as methanol/water [38,39].

While ethanol, methanol, and their mixtures with water often provide the best yields for extracting plant polyphenols, other solvents have also been frequently employed. [40]. Henanou and Zaghez, 2019 showed, in their work, that the extraction yield depends on the extraction method and thus on the choice of solvents used and their physicochemical characteristics, in particular their polarity [41].

These results led to deduce that the extraction of phenolic compounds by Soxhlet remains the best method to obtain good performance because it promotes the relatively complete extraction of the metabolites present in the plant. Again, the extraction yield mainly depends on the polarity of the solvent.

### 3.4. Polyphenol Content

**Figure 4:**
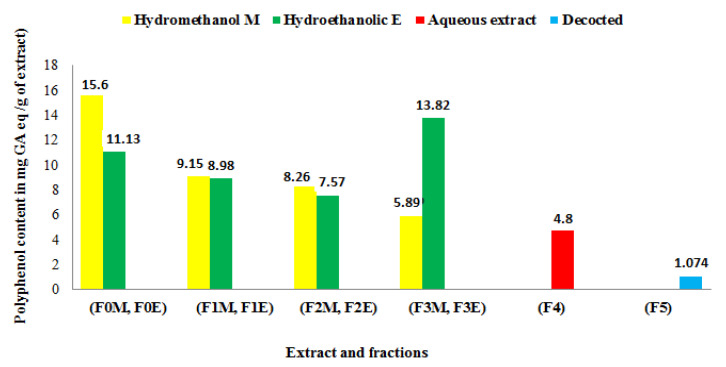
Polyphenol content of *R. segetum* (L.) Moris extracts expressed in milligrams of gallic acid equivalents per gram of extract (mg eq FA/g).

Each extract’s total phenol content was reported in milligrams (mg) gallic acid equivalent per gram (g) of dried plant material or extract, as determined using the Folin-Ciocalteu technique. The formula for the linear regression of the gallic acid calibration curve is: y = 0.095x + 0.003 with a correlation coefficient R2 = 0.998. Figure 4 summarizes the results, obtained by a UV-visible spectrophotometer, relating to the total phenol contents of the crude hydrometanol (F_0M_) and hydroethanol (F_0E_) extracts, the ethyl acetate fractions (F_1M_, F_1E_), the butanols (F_2M_, F_2E_) and the aqueous fractions (F_3M_, F_3E_), the aqueous extract (F_4_) and the decoction (F_5_). The results reported in Figure 4 show that the high content of total phenols was found in the crude hydromethanolic extract (15.6 ± 1. 76 mg EAG/g) followed by the aqueous fraction of the hydroethanolic extract (13.82 ± 1.88 mg EAG/g of extract). While the extract obtained by decoction is less rich in total phenols (1.078 ± 1. 75 mg EAG/g of extract).

Analysis of the collected data reveals that the polyphenol content of the plant extracts under study varies with the polarity of the solvent employed and the extraction technique. Thus, it may be inferred that polar solvents were superior to less polar solvents in extracting phenolic chemicals from the plant [42].

The difference in phenol content of the extracts of the same plant depends, in addition to extraction solvent, on several factors such as the environmental conditions of the plant (altitude, temperature, light) as well as soil conditions and ripening state of the plant.

### 3.5. Flavonoid Content

The amount of flavonoids present in each extract was reported as the amount of quercetin equivalent per milligram of dry plant material (mg EQ/g) using the aluminum trichloride technique. The calibration curve is established using quercetin, the linear regression formula of the quercetin calibration curve is y = 0.075x − 0.003 with a correlation coefficient R_2_ = 0.995.

**Figure 5:**
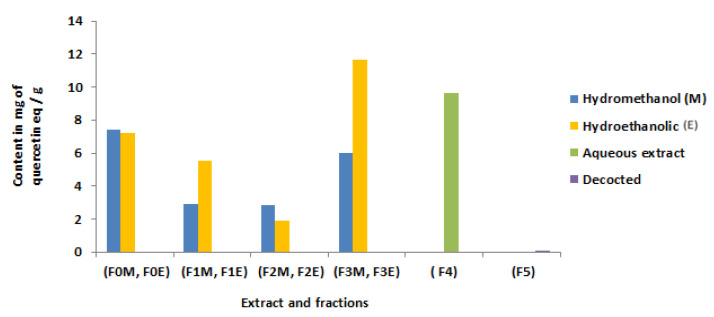
Flavonoid content of *R. segetum* (L.) Moris extracts in mg of quercetin/g.

The results show that the aqueous extract, the hydroethanolic and hydromethanolic extracts of *R. segetum* (L.) Moris contain greater amounts of total flavonoids compared to the decoction. The residual phase of the hydroethanolic extract has a high content of flavonoids (11.63 mg EQ/g) followed by the aqueous extract 9.66 mg EQ/g) (Figure 5).

### 3.6. Condensed Tannin Content

A calibration curve is established using (+)-catechin as a reference. The formula for the linear regression of this curve is 𝑦 = 0.7421𝑥 + 0.0318 with a correlation coefficient R^2^ = 0.9978. The condensed tannin contents are determined from the linear regression equation of this curve expressed in mg equivalent of (+)-catechin per g of dry matter or extract.

**Figure 6:**
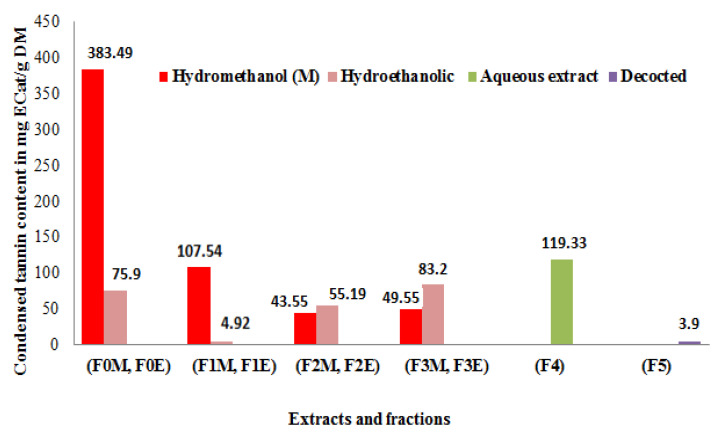
Condensed tannin content of *R. segetum* (L.) Moris extracts in mg ECat/g DM.

According to the results presented in Figure 6, it is clear that the extracts of *R. segetum* (L.) Moris have a high condensed tannin rate of the order of 383.49 mg ECat/g of DM for the crude hydromethanolic extract followed by the aqueous extract 119.33 mg ECat/g of DM; 107.54 mg ECat/g DM for the ethyl acetate fraction of the F_1M_ hydromethanolic extract. The decocted extract has a lower content than those found in the other extracts for Soxhlet extraction, it is around 3.9 mg ECat/g of dry matter. These results allowed us to deduce that Soxhlet extraction remains the best method to obtain the highest contents of condensed tannins and the hydromethanolic mixture is the best extraction solvent.

We conclude that the different phases do not have the same richness in condensed tannins, which suggests that some solvents are better suited than others for the extraction of its compounds.

### 3.7. Identification and Quantification of the Polyphenols Contained in the Extract of R. segetum *(L.)* Moris by High-Pressure Liquid Chromatography Coupled with UV Spectrometry (HPLC/DAD)

**Figure 7:**
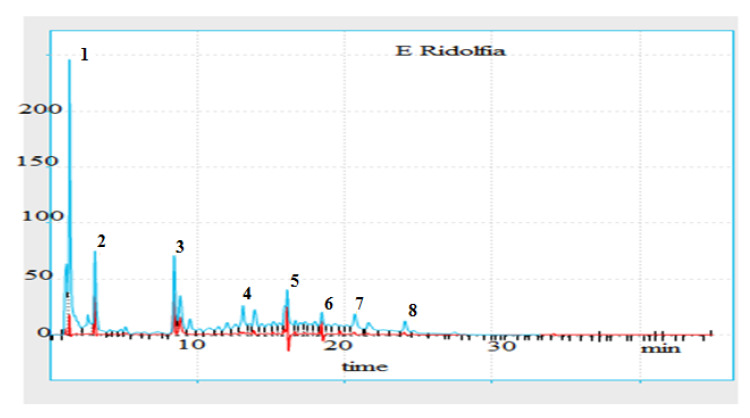
Chromatogram of the decocted extract of *R. segetum* (L.) Moris by HPLC-DAD.

Analysis by high-performance liquid chromatography (HPLC) coupled with detection methods (UV spectrophotometry) of decoction of *R. segetum* (L.) Moris makes it possible to deduce important information on their chemical compositions. The chromatographic conditions used made it possible to obtain a chromatogram with well-separated peaks (Figure 7).

**Figure 8:**
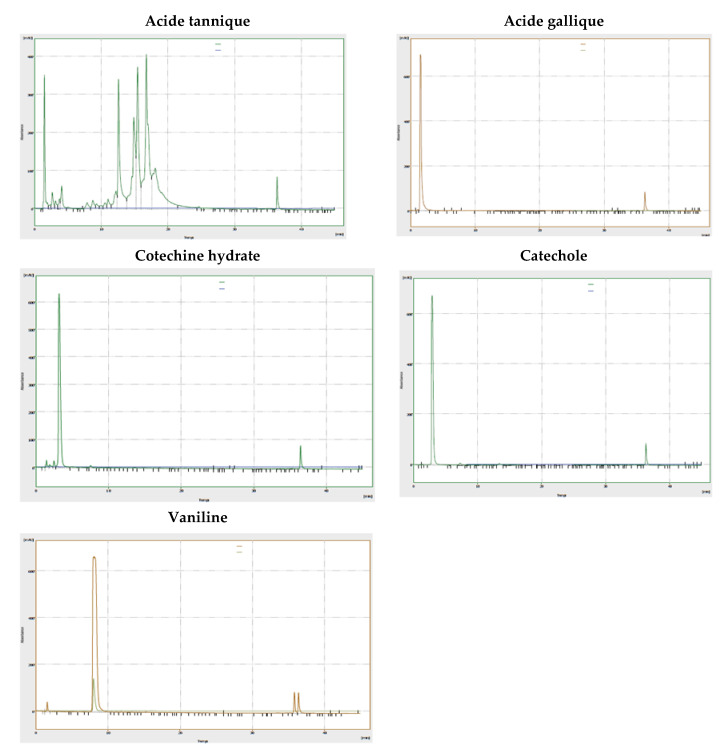
Chromatogram of standards by HPLC-DAD.

The comparison of the retention times of these peaks and those of the various available standards (Figure 8) revealed the presence of 8 phenolic compounds. So, to be sure that it is indeed the identified molecule, we overloaded the extract with standards corresponding to the identified molecules. These are gallic acid, catechol, vanillic acid, catechin, tannic acid, rosmarinic acid, naringenin and coumarinic acid (Table 5).

We were able to identify eight polyphenols and the total percentage identified is 52.6% (Table 5): Including flavonoids such as naringenin, tannins such as tannic acid and catechin, phenolic acids such as gallic acid, catechol, vanillic acid, rosmarinic acid and coumarinic acid. These results therefore confirm the colorimetric analysis of this extract.

After quantification, we found that the majority molecule is gallic acid, which can contribute to the nutritional value and medicinal properties of the plant. Hence the justification of its various biological properties such as antioxidant activity [5,6]; antibacterial activity [6,12,13,14], anti-inflammatory activity [5], anticancer activity [15,16], HIV-1 inhibition [4] and insecticidal activity [14,17].

### 3.8. Antioxidant Activity

#### 3.8.1. DPPH* Free Radical Trapping Method

To compare the antioxidant capacity of *R. segetum* (L.) Moris and the standard antioxidant (ascorbic acid) about the DPPH* radical was examined using a spectrophotometer by tracking the reduction of this radical which is accompanied by its shift from the violet color (DPPH*) to the yellow color (DPPH-H) measured at 515 nm. Absorbance changes brought on by the presence of antiradical compounds are used to quantify this potential for reduction [43]. 

##### Determination of Inhibition Percentages

**Figure 9:**
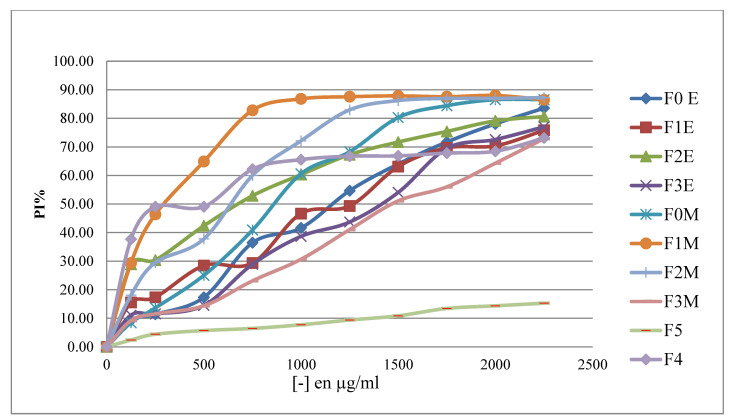
Percentages of DPPH* inhibition according to the concentrations of the extracts and the fractions extract of *R. segetum* (L.) Moris.

The results of the percentage of the antioxidant activity of the hydrometanolic (F_0M_) and hydroethanolic (F_0E_) crude extracts, the ethyl acetate fractions (F_1M_, F_1E_), the butanol fractions (F_2M_, F_2E_) and the aqueous fractions (F_3M_, F_3E_), the aqueous extract (F_4_) and the decoction (F_5_) of aqueous *R. segetum* (L.) Moris against the free radical DPPH are illustrated in Figure 9. We observe, according to Figure 8, that the increase in the concentration of different extracts and of ascorbic acid leads to an increase in the anti-radical activity, then the greater the concentration of the extracts, the greater the quantity of DPPH˙ free radicals in the reaction medium is small.

##### Determination of Inhibition Percentages

The results of the percentage of the antioxidant activity of the hydrometanolic (F_0M_) and hydroethanolic (F_0E_) crude extracts, the ethyl acetate fractions (F_1M_, F_1E_), the butanol fractions (F_2M_, F_2E_) and the aqueous fractions (F_3M_, F_3E_), the aqueous extract (F_4_) and the decoction (F_5_) of aqueous *R. segetum* (L.) Moris against the free radical DPPH are illustrated in Figure 9. We observe, according to Figure 8, that the increase in the concentration of different extracts and ascorbic acid leads to an increase in the anti-radical activity, then the greater the concentration of the extracts, the greater the quantity of DPPH˙ free radicals in the reaction medium is small.

**Figure 10:**
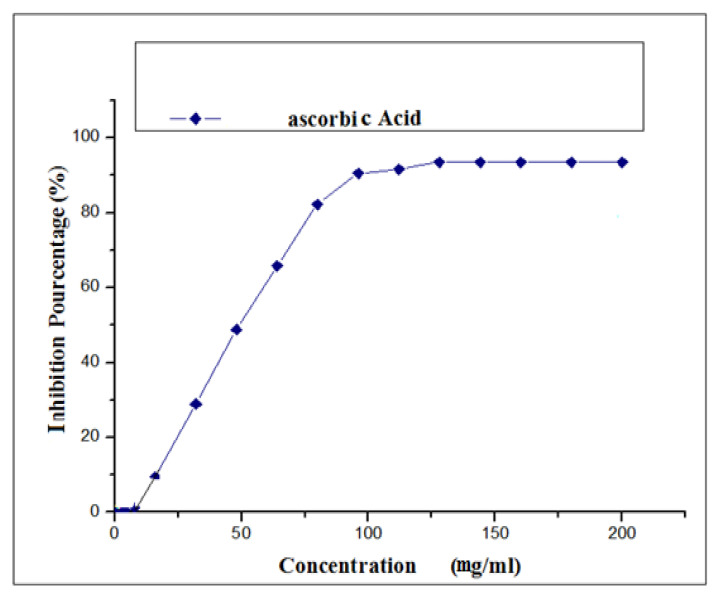
Percentages of DPPH*inhibition according to the concentrations in mg/mL of ascorbic acid.

Figure 10 shows that ascorbic acid, a well-established positive control due to its potent antioxidant action, has a linear relationship with the percentage of free radical inhibition. Regarding the anti-radical activity of the extracts, we noted that the crude hydromethanolic extract reached 87.26%; while the crude hydroethanolic extract records 83.56%, as for the aqueous and decocted extracts, they respectively reach the values of 73.16% and 15.29%. However, these values remain lower than that of ascorbic acid whose maximum value is of the order of 90.49%.

##### Determination of Inhibitory Concentration IC_50_

**Figure 11:**
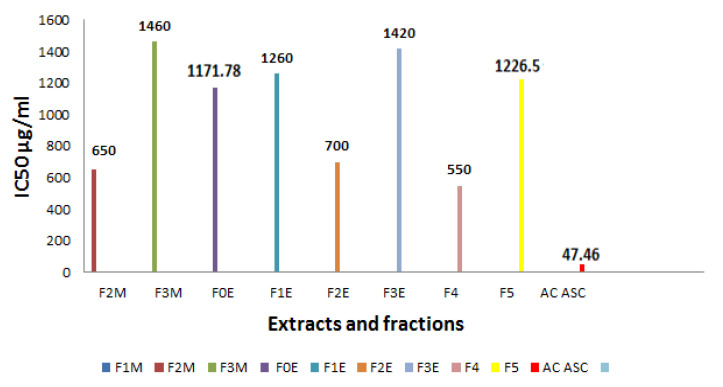
IC50 of extracts of *R. segetum* (L.) Moris and ascorbic acid (AC AUC) (in µg/mL).

We determined the IC_50_ parameter, which is defined as the concentration of substrate (extract) required to reduce 50% of the initial concentration of DPPH (Figure 11). This graphically determined concentration is expressed in μg/mL. The lower the IC_50_ value, the more the extract is considered a powerful anti-free radical [44].

According to the results recorded in Figure 11, the CI_50_ of the various extracts and fractions tested of *R. segetum* (L.) Moris is compared with that of ascorbic acid, we notice that the anti-radical activity of all our extracts is lower than the capacity scavenging of the DPPH˙ radical of the substance reference whose IC_50_ = 47.46 µg/mL. This capacity is greater in the aqueous extract (F_4_) followed by the ethyl acetate fractions (F_2M_, F_2E_) and *R. segetum*, their IC_50_ respectively are 550 µg/mL, 650 µg/mL, 700 µg/mL.

The high concentration of phenolic compounds in the crude extracts is responsible for their potent anti-free radical action (compounds phenolics, flavonoids, and tannins). Findings suggest that the plant’s ethyl acetate fractions (FEM, F3M) have potent anti-free radical properties. These extracts have a high ac-tivity because they are abundant in phenolic chemicals, which contain the most molecules per unit of dosage (phenolic compounds, flavonoids and condensed tannins). A rise in anti-radical activity was seen in plant extracts, according to research by Kang et al., who hypothesized that this was due to the presence of polar molecules [45].

#### 3.8.2. Iron Reduction by *R. segetum* (L.) Moris Extracts

##### Evaluation of Iron Reduction Capacity by Extracts and Fractions of *R. segetum* (L.) Moris

**Figure 12:**
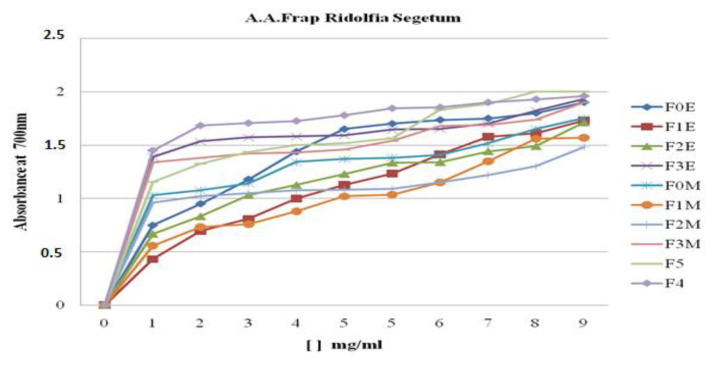
Reducing power of *R. segetum* (L.) Moris extracts measured by iron method.

**Figure 13:**
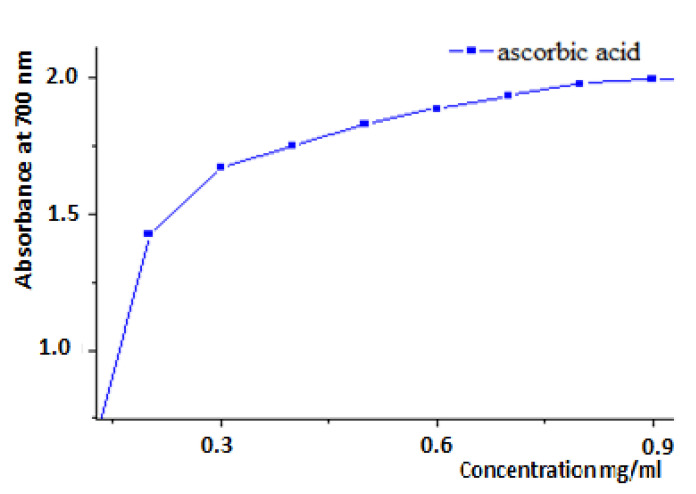
Reducing power of ferric iron by ascorbic acid.

The reducing activity of ferric iron of yellow color, to ferrous iron of blue-green color, was carried out on the hydrometanolic (F_0M_) and hydroethanolic (F_0E_) crude extracts, the ethyl acetate fractions (F_1M_, F_1E_), the butanol fractions (F_2M_, F_2E_) and the aqueous fractions (F_3M_, F_3E_), the aqueous extract (F_4_) and the decoction (F_5_) of *R. segetum* (L.) Moris (Figure 12) as well as the ascorbic acid (Figure 13), standard used as reference. The values obtained made it possible to plot the curves represented in Figure 11. The results obtained showed that the reducing capacity of iron is proportional to the increase in the concentration of the samples studied.

##### Determination of the Effective Concentration (EC_50_) of Extracts of *R. segetum*

**Figure 14:**
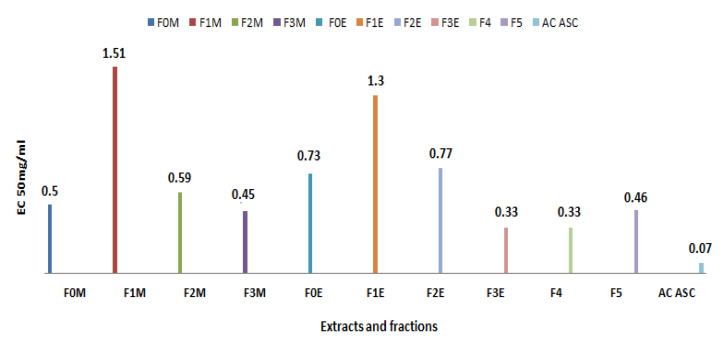
Effective concentrations EC50 of extracts of *R. segetum* (L.) Moris measured by the iron method.

The antioxidant capacity of the different extracts is expressed by determining the effective concentration (EC_50_) which corresponds to an absorbance equal to 0.5 in order to compare their reducing activities. The values are shown in Figure 14.

Based on the data, we can see that the ability of the various extracts investigated to decrease iron varies; the aqueous fraction (F3E) and the aqueous ex-tract (F4) have the highest CE50 = 0.33 mg/ml, followed by the residual fraction (F3M) and the decoc-tion, which have an EC50 capacity = 0.4 mg/ml. Therefore, all extracts have some iron-reducing capacity, but not as much as ascorbic acid (EC_50_ = 0.07 mg/mL).

The results obtained show that all the extracts tested are characterized by a very pronounced increase in iron reduction as a function of the increase in the concentration which presented the most activity in terms of iron reduction.

### 3.9. Antihyperglycemic Effect of Decocted Extract of R. segetum *(L.)* Moris

#### 3.9.1. Acute Safety of *R. segetum* (L.) Moris Decoction

When a drug is administered to a live organism, either at a relatively large single dosage or at low doses repeatedly over a long period of time, it may generate toxic consequences, which can include both morphological and functional defects. Toxicology refers to the battery of pharmacological tests used to establish the nature and extent of a substance’s toxicity in order to control its distribution and administration. This acute toxicity test, performed in vivo on mice, demonstrates that the decoction is nontoxic at 2 g/Kg. Throughout the whole of the monitoring period, neither toxicity symptoms (such as nausea, vomiting, and loss of appetite) nor fatality rates were seen.

Considering its culinary uses, we can assume that *R. segetum* (L.) Moris has negligible adverse effects. The use of *R. segetum* (L.) Moris in traditional Moroccan medicine has shown that there are no significant adverse effects associated with the consumption of this plant. Miranda et al. reported that *R. segetum* (L.) Moris non-toxic [46]. The species has been reported to be eaten raw in salads, these cleaned and peeled stems are eaten [3]. The infusion of the plant is used as a stomachic and the pulverized fruits of *R. segetum* are decocted with madder and an egg [47].

#### 3.9.2. Antihyperglycemic Effect of *R. segetum* (L.) Moris Extract in Normal Rats

**Figure 15:**
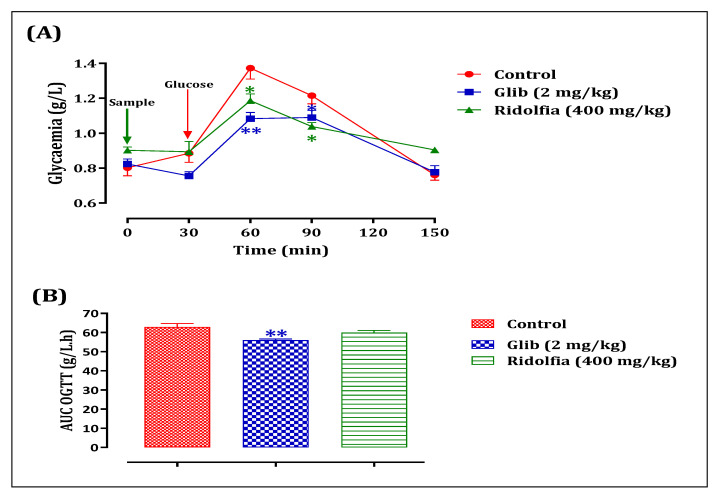
Variation in postprandial glycemia (**A**) and area under the postprandial glycemia curve (**B**) in normal rats after administration of the test products (decoction and glibenclamide). Values are means ± SEM. (n = 6). ** *p* < 0.01; * *p* < 0.05: in comparison with the control.

Normal rats given 400 mg/kg of the extract orally 30 min before a glucose overload had significantly less postprandial hyperglycemia at 60 min (*p* less than 0.05; 1.186 0.04 g/L), 90 min (p0.05; 1.038 0.15 g/L), and 150 min (p0.05; 0.904 0.04 g/L) than rats given distilled water first. At 60 min (1.3733 0.08 g/L), 90 min (1.215 0.09 g/L), and 150 min (0.76 0.07 g/L), glucose overload caused very high blood sugar. Also, glibenclamide stopped postprandial hyperglycemia by a lot in the 150 min after a glucose overload, at 60 min (*p* less than 0.001; 1.083 0.04 g/L), 90 min (p0.05; 1.09 0.03 g/L), and 150 min (0.7766 0.06 g/L) compared to the group of rats that had been given distilled water before the glucose overload (Figure 14A). The area under the curve (AUC glucose) was also significantly (*p* less than 0.01) lower in rats given the extract than in rats given distilled water (59.91 2.58 g/L/h vs. 62.91 4.32 g/L/h). Also, the area under the curve of glibenclamide was significantly (*p* less than 0.01) lower (55.95 1.69 g/L/h) than the area under the curve of rats treated with dis-tilled water (62.91 4.32 g/L/h) (Figure 15B).

The results of our research showed for the first time that the extract could improve D-glucose tolerance by lowering blood sugar levels after a meal. In fact, giving the extract to healthy rats by mouth had a big effect on lowering their blood sugar. D-glucose was too much. In addition, the extract stopped D-glucose from being absorbed in the gut. D-glucose couldn’t get from the gut to the bloodstream because of the extract.

#### 3.9.3. Evaluation of the Inhibitory Effect of Aqueous Extracts on the Activity of Pancreatic α-Amylase, In Vitro

**Figure 16:**
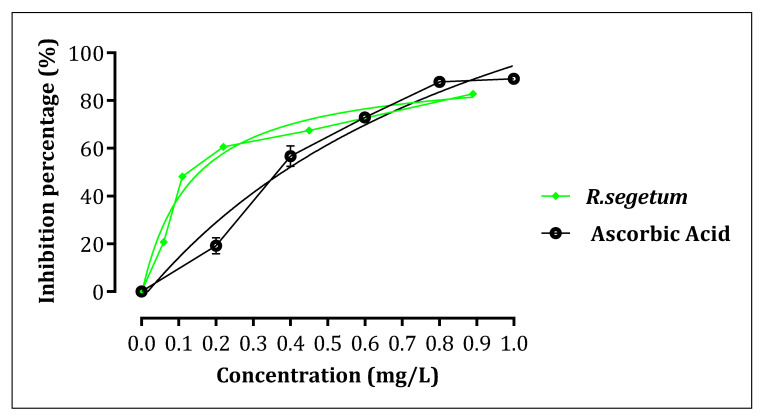
Inhibitory effect on α-amylase activity by *R. segetum* (L.) Moris extract and ascorbic acid, in vitro. Values are means ± SEM.

The results of the effect of *R. segetum* (L.) Moris extract on pancreatic α-amylase activity, in vitro, is shown in Figure 16. Indeed, pancreatic α-amylase enzyme activity was significantly inhibited in the presence of the *R. segetum* (L.) Moris extract (IC_50_ = 0.133 ±0.09 mg/mL) in comparison with the control test. Moreover, this inhibiting effect of α-amylase by the extract of *R. segetum* (L.) Moris is lower than that of ascorbic acid (IC_50_ = 1.046 ±0.01 mg/mL). Our result showed that the decoction exerts a dose-dependent inhibitory effect against pancreatic α-amylase. Studies have suggested that inhibiting enzymes involved in sugar digestion, such as intestinal α-glucosidase and pancreatic α-amylase, is one of the treatments used to control high blood sugar in people with diabetes. To delay and reduce the intestinal absorption of D-glucose [48,49].

The extract of *R. segetum* (L.) Moris is rich in phenolic compounds (phenolic acids, flavonoids and tannins) which justify our important results. These compounds have the property of inhibiting the key enzymes of carbohydrate metabolism, α-glucosidase, and α-amylase [50,51]. 

As a result, these phenolic compounds are distinguished by high levels of antioxidant activity and are well-known for their antidiabetic action, which enables the management of the disturbed oxi-dant environment present in diabetic circumstances [52,53]. Reports have shown that antioxidants protect L6 muscle cells’ sensitivity to insulin [54]. In-depth chemical analysis of this extract has shown that it contains gallic acid. Gallic acid has been linked in several studies to the suppression of pancreatic –amylase [55,56,57]. 

## 4. Conclusions

This study allowed us to deduce that *R. segetum* (L.) Moris is an aromatic and medicinal plant rich in molecules with therapeutic potential. Also, the HPLC-DAD chromatographic analysis confirmed the richness of the species in polyphenols. In particular phenolic acids, flavonoids and tannins are natural substances of considerable interest in pharmacology. To manage diabetes mellitus, it is necessary to precisely control glucose levels after meals, using plant extracts that have an antihyperglycemic effect. The decoction of *R. segetum* showed an anti-hyperglycemic effect thanks to its richness in side metabolites with ant hyperglycemic effects and an important antioxidant potential. Besides beneficial effect, that this plant was not toxic. Moreover *R. segetum* (L.) Moris extract inhibits pancreatic α-amylase enzymatic activities. Furthermore, enhanced *R. segetum* (L.) Moris enhances in vivo antihyperglycemic effect in rats after acute oral administration.

These results confirm the traditional use of *R. segetum* (L.) Moris in the treatment of type 2 diabetes. In light of our results, we can conclude that *R. segetum* (L.) Moris has a high potential antihyperglycemic. Thus, this could justify their use in traditional medicine for the management of diabetes.

## Figures and Tables

**Table 1:** Systematic classification of *R. segetum* (L.) Moris.

Superdivision	Spermatophyte
Subclasse	Rosidae
Order	Apials
Branch line	Spermaphyte (phanerogam)
Branch	Angiosperme
Class	Dicotyledon
Subclass	Dialypetal (with separate petals)
Order	Apiale (ombellale)
Family	Apiaceous (Umbelliferous)
Genre	*Ridolfia*
Species	*Segetum*

**Table 2:** Moisture Content (TH), Ash and pH of *R. segetum* (L.) Moris.

Speces	TH (%)	MS (%)	pH	MO (%)	Ash (%)
*R. segetum*	12.49 ± 0.01	91.8	5.21	2.18 ± 0.15	97.82 ± 0.15

**Table 3:** ICP mineral composition of *R. segetum* (L.) Moris.

Elements	Mn	Cu	Cr	Zn	As	Pb	Cd	Sb	Al	Ca	Fe	Ti
En(mg/L)	0.1359	0.0332	0.0041	0.0585	0.0319	0.0344	ND	0.0208	0.4799	47.685	2.358	0.0166

ND: undetectable.

**Table 4:** Results of phytochemical screening of extracts of *R. segetum* (L.) Moris.

Chimical Groups			Reagents/Reaction	*Ridolfia segetum*
Alcaloïds			Valse-Mayer Reagents	+
			DragendorffReagents	+
Polyphénols	total Tannins	T Gallic tannins	Stiasny HCl Reagents	+
		Catechic tannins		-
	Anthocyanes		Acid-base reaction	-
	Free flavonoids		Reaction to cyanidin with Mg	+ +
	Leukocytes		Reaction to cyanidin without Mg	+ ++
Free anthracene derivatives			Bornträger Reaction	-
Combined Santhracenoic Derivatives		O-heterosides	Coloration Reaction	+ +
		C-heterosides		
Sterols et triterpenes			Libermann-Burchard Reaction	++ +
Mucilages			Precipitation Reaction	+ ++
Saponosides			Foam test	+

(-): absence; (+): presence; (++): abundance.

**Table 5:** List of compounds identified using (HPLC/DAD) in the decocted extract of *R. segetum* (L.) Moris.

*Phenolic Compounds*	TR (Min)	Percentage %	Metabolite Families		
		*R. segetum*	Phenolic Acid	Flavonoids	Tannins
Gallic Acid	1.43	25.2	+	-	-
Cathechol	2.9	7.7	+	-	-
Vanillic Acid	8.05	7.3	+	-	-
Catechin	12.27	2.2	-	-	+
Tannic Acid	16.76	4	-	-	+
Rosmarinic Acid	18.93	1.1	+	-	-
Naringenin	22	1.2	-	+	-
Coumarinic Acid	24.1	1.3	+	-	-

## Data Availability

Not applicable.
